# Parental Choices Following Prenatal Diagnosis and Neonatal Mortality Trends: A Population‐Based Analysis

**DOI:** 10.1111/1471-0528.18284

**Published:** 2025-07-04

**Authors:** Nicolas Bourgon, Elsa Kermorvant‐Duchemin, Philippe Roth, Alexandre Lapillonne, Yves Ville

**Affiliations:** ^1^ Obstetrics, Fetal Medicine Surgery and Imaging Unit Université Paris Cité, AP‐HP, Hôpital Necker‐Enfants Malades Paris France; ^2^ Department of Neonatal Medicine Université Paris Cité, AP‐HP, Hôpital Necker‐Enfants Malades Paris France

**Keywords:** delivery: perineal care, fetal diagnosis and therapy, health economists, paediatrics: neonatal, palliative care, statistics: epidemiological surveys

The neonatal period, the first 28 days of life, represents a critical window of vulnerability, accounting for 46% of all under‐five deaths worldwide [1]. The neonatal mortality rate (NMR), expressed per 1000 live births (LB) and encompassing both early (days 0–6) and late (days 7–27) deaths, is a key indicator of maternal and child health and of quality of perinatal care. In Europe, the NMR declined significantly by 18% between 1996 and 2005, whilst significant regional disparities persist [2]. As in other high‐income countries, the leading causes of neonatal mortality include congenital anomalies (CAs), affecting approximately 25 per 1000 births in Europe, as well as complications related to preterm birth and perinatal injuries [1]. Despite a decline in infant mortality due to congenital anomalies in Europe, CAs are reported as the leading recorded cause of neonatal death, accounting for up to 71% of cases in some dataset based on heterogeneous and unstandardized methods [3]. The reasons behind this decline remain complex and unclear. It is still uncertain whether it is primarily driven by advancements in perinatal care for neonates and expectant mothers, an increasing stillbirth rate as part of the natural progression of certain conditions, or a reduction in NMR due to terminations of pregnancy following prenatal diagnosis.

Contrary to the general downward trend observed in other high‐income countries, France has experienced a concerning rise in NMR in recent years. Initially, this increase was driven by a rise in late neonatal mortality between 2005 and 2012, followed by a marked surge in early neonatal mortality since 2012 [4]. Simultaneously, an increasing number of pregnancies affected by severe and incurable fetal conditions, which are legally eligible for termination, are now being carried to term following prenatal diagnosis (Figure 1). According to data from the French National Biomedical Agency, the incidence of such cases has indeed risen from 0.60 to 2.68 per 1000 LB between 2008 and 2022. Among these newborns, the NMR may have doubled from 0.2 to 0.4 per 1000 LB over the same period, potentially contributing to the rise in neonatal mortality (Figure 1). Supporting these trends, recent data show that approximately 13% of newborns with severe and incurable fetal conditions received perinatal palliative care with limitations of life‐sustaining treatment, with decisions made either during pregnancy or following neonatal assessment [5]. If neonatal mortality statistics were adjusted to exclude newborns with severe and incurable fetal conditions whose pregnancies were continued, the overall NMR in France would appear relatively stable, decreasing slightly from 2.4 per 1000 LB in 2008 to 2.3 per 1000 LB in 2022 (Figure 1).

**FIGURE 1:**
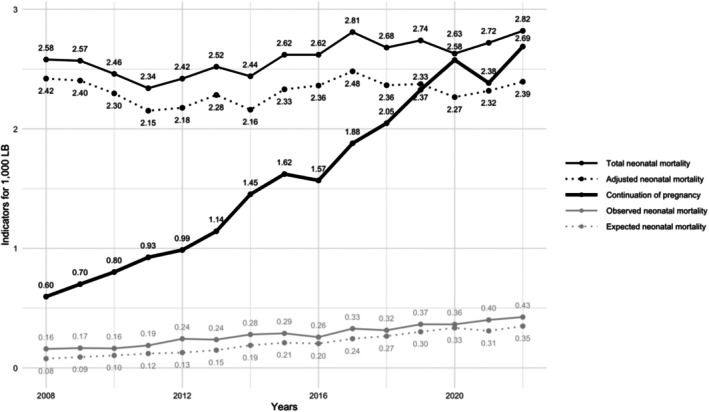
Evolution of indicators (rates per 1000 LB) in France between 2008 and 2022. Data from the French National Institute of Statistics and Economic Studies (INSEE, https://www.insee.fr/fr/accueil) and the French National Biomedical Agency https://www.agence‐biomedecine.fr. Black solid curve: Total neonatal mortality. Black dotted curve: Adjusted neonatal mortality, excluding deaths in newborns with severe and incurable fetal conditions. Thick black solid curve: Pregnancies carried to term following the prenatal diagnosis of severe and incurable fetal conditions. Grey solid curve: Neonatal mortality in newborns with severe congenital conditions. Grey dotted curve: Expected neonatal mortality related to severe and incurable fetal conditions (estimated cases for perinatal palliative care with life‐sustaining treatment limitation).

Parental choices regarding pregnancy continuation are increasingly shaped by societal changes, including advancements in neonatal care and evolving perceptions of disability. These changes must be considered in any comprehensive analysis of neonatal and infant health, as they play a crucial role in shaping outcomes. Accounting for these changes is essential when designing population‐based interventions and evaluating their long‐term impact and sustainability [6]. Moreover, this shift in parental decision‐making regarding pregnancy continuation raises important questions about long‐term infant outcomes. Many of these children will experience significant morbidity, often resulting in severe or lifelong disabilities. Recognising and accounting for these societal factors will enable policymakers to make more informed decisions about the future organisation and capacity of healthcare systems. A long‐term, strategic approach is needed to ensure adequate infrastructure, effective resource allocation, and sustained support for both affected children and their families.

## Author Contributions

Nicolas Bourgon contributed with conceptualisation, investigation, writing – original draft, writing – review and editing. Elsa Kermorvant, Philippe Roth, Alexandre Lapillonne, and Yves Ville contributed to writing – review and editing.

## Conflicts of Interest

The authors declare no conflicts of interest.

## Data Availability

The data used for this study are openly accessible on the website of the French National Institute for Statistics and Economic Studies (INSEE, https://www.insee.fr/fr/accueil) and the French National Biomedical Agency (https://www.agence‐biomedecine.fr).
